# Association Between Anthropometric Measurements and Mediterranean Lifestyle in Women Diagnosed with Hashimoto’s Thyroiditis: Data from the Mediterranean Region

**DOI:** 10.3390/nu17050892

**Published:** 2025-03-03

**Authors:** Burcin Karavelioglu, Taygun Dayi, Osman Koseoglulari, Adile Oniz

**Affiliations:** 1 Department of Nutrition and Dietetics, Faculty of Health Sciences, Near East University, Mersin 99000, Turkey; burcin.karavelioglu@neu.edu.tr; 2 Unit of Nutrition and Dietetics, Near East University Hospital, Mersin 99000, Turkey; 3 Marmara Clinic, Mersin 99000, Turkey; marmaradiyabet@hotmail.com; 4 Department of Neurosciences, Institute of Graduate Studies, Near East University, Mersin 99000, Turkey; adile.oniz@neu.edu.tr; 5 Brain and Conscious States Research and Application Center, Near East University, Mersin 99000, Turkey

**Keywords:** Mediterranean diet, Hashimoto’s thyroiditis, Mediterranean lifestyle, anthropometric measurements

## Abstract

Background/Objectives: Hashimoto’s thyroiditis (HT) is the most common autoimmune disease which lowers a patient’s quality of life. Our study aimed to assess the association between Mediterranean lifestyle and anthropometric measurements in patients with HT. Methods: This study was conducted with 120 female patients previously diagnosed with HT. The ‘Mediterranean Diet Adherence Screener-(MEDAS)’ and ‘MEDiterranean LIFEstyle-(MEDLIFE)’ scales were used to assess diet quality and lifestyle. Also, some anthropometric measurements were collected. Spearman’s Correlation Test was used to determine correlations between two quantitative variables. Results: The average age of the participants was 37.49 ± 7.47 years. The average diet quality scores were 7.80 ± 1.93 for MEDAS and the total life quality score was 16.41 ± 3.74 for MEDLIFE. Significant negative correlations were observed between the MEDLIFE total score and hip and waist circumferences, body weight, and body fat (%) (*p* < 0.05), as well as with the score of physical activity, rest, social habits, and conviviality (*p* < 0.05). Furthermore, an increase the Mediterranean dietary habits score was associated with a decrease in these measurements (*p* < 0.05). Conclusions: The MEDLIFE score, beyond only the Mediterranean dietary habits sub-score, was found to be associated with lower anthropometric measurements in patients with HT, who are at higher risk of pre-obesity and obesity.

## 1. Introduction

Hashimoto’s thyroiditis (HT) is a common autoimmune disease which affects women 7–10 times more often than men [1]. It is characterized by high levels of thyroid antibodies. Specifically, it is marked by elevated levels of anti-TPO (thyroid peroxidase) and anti-Tg (thyroglobulin) antibodies in serum, and it is influenced by many factors [2,3]. A recent comprehensive population survey on this Mediterranean island reported that 51.7% of the participants had at least one chronic disease, 8.4% had thyroid diseases, and thyroid diseases were more common in women (82.3%) [4]. A general study conducted in Greece, a Mediterranean country, reported a thyroid disease prevalence of 9%, with 8.6% being hypothyroidism, and 14.9% of these patients identified as women [5].

Thyroid diseases have been among the most common chronic conditions in individuals with multi-morbidity. Disease combinations most often involve the circulatory and endocrine systems especially due to weight gain, accounting for 25% of cases [6]. Many studies in the current literature proposed an association between HT and obesity risk. Some of these studies underlined that pre-obesity and obesity prevalence are high in patients with HT [7,8,9].

The Mediterranean lifestyle, shaped by the traditional lifestyle and nutrition of Mediterranean countries such as Spain, Italy, Croatia, and Greece, is considered the healthiest lifestyle model today. This lifestyle, which includes moderate physical activity, adequate rest, quality sleep, and the environmentally friendly food sources, emphasizes the daily consumption of vegetables, fruits, whole grains, nuts, and olive oil prepared with local and seasonal foods. Limiting the consumption of animal products, encouraging the consumption of seafood and white meat instead of red meat, restricting the consumption of refined sugar, and moderate consumption of red wine are all recommended [10,11]. Compliance with the Mediterranean lifestyle can regulate thyroid function, strengthen the immune system, and perhaps most importantly, reduce thyroid antibodies [12,13]. Merchan-Ramirez et al. (2024) emphasized that not only food intake but also adherence to the Mediterranean diet, together with good sleep quality and moderate physical activity, which is defined as the Mediterranean lifestyle, can influence thyroid-stimulating hormone (TSH) and Thyroxine (T4) levels [13]. In addition to the beneficial effects of the prognosis of the disease, adherence to the Mediterranean diet and lifestyle can reduce pre-obesity and obesity risk in patients with HT according to the current studies [12,14].

From this perspective, this study aims to explore the association between adherence to the Mediterranean lifestyle and obesity-related anthropometric measurements among women diagnosed with HT.

## 2. Materials and Methods

### 2.1. Study Setting

This study was conducted face-to-face in the country’s largest endocrinology and metabolism disorders clinic which is frequently visited by women of childbearing age residing in Northern Cyprus who have been diagnosed with HT and are experiencing endocrine-related health issues. To determine the sample size, the number of patients diagnosed with HT per month (including only patients compliant with this study, e.g., female patients aged 19–55 years old) in the Marmara clinic was requested. Patient appointments were recurring every six months. The total number of HT-diagnosed patients who visited the health center six months before the onset of the study was 175. With this in mind, in this study, the sample size was calculated using the ‘G. Power-3.1.9.2’ program at a 95% confidence level. As a result of the analysis, with α = 0.05, a standardized effect size of 0.3 (medium effect size), and a theoretical power of 0.95, the minimum sample size was calculated to be 138. All HT-diagnosed patients in this clinic were invited to participate in this study. However, only 120 of them voluntarily participated (86.95% of the calculated sample size).

### 2.2. Ethical Compliance of the Research

The ethical compliance of the study was ensured through the submission of a detailed project report to the Near East University Scientific Research Ethics Board, which was approved by the board members on 21 June 2023 (Project number: YDU/2023/115-1749).

### 2.3. Study Plan and Data Collection Tools

In this study, data were collected utilizing the Mediterranean Diet Adherence Screener (MEDAS) and the Mediterranean Lifestyle Index (MEDLIFE). Additionally, anthropometric measurements of all participants were recorded. Participants’ personal information, including their age, thyroid gland-related health conditions, overall health status, and nutritional habits, was collected.

### 2.4. Mediterranean Diet Adherence Screener—MEDAS (Mediterranean Diet Adherence Scale)

This scale was developed to assess adherence to the Mediterranean diet in adults and consists of 14 items. There are two response choices: ‘yes’ or ‘no’. Each Mediterranean diet-compliant response is awarded +1 point [15]. Scoring is based on the total score, with ≤6 points indicating ‘low’, 7–8 points indicating ‘moderate’, and ≥9 points indicating ‘high’ adherence to the Mediterranean diet [16]. The Turkish validity and reliability study of MEDAS was conducted by Pehlivanoglu et al. in 2020, and it was deemed to be suitable for use in the Turkish population [17].

### 2.5. MEDiterranean LIFEstyle (MEDLIFE)

The MEDiterranean LIFEstyle (MEDLIFE) scale consists of 28 items and three sections. The first section contains 15 items and evaluates the level of adherence to Mediterranean-style food consumption. Each item has response compliant with Mediterranean-style food consumption. Providing this response earns the respondent +1 point. The second section includes seven questions pertaining to Mediterranean eating habits (consumption of salt, sugar, refined products, etc.). All questions, except question 16, have ‘yes’ or ‘no’ response choices. Each ‘yes’ response is worth +1 point. Also, question 16 requires a response compliant with Mediterranean eating habits, which is worth +1 point. The last section includes six questions about physical activity, sleeping patterns, resting habits, and time spent on social activities and entertainment. The first two questions are each worth +1 point if the response is compliant with Mediterranean-style daily life activities, and others are worth +1 point if the response is ‘yes’. There is no classification of MEDLIFE according to total or sub-scores. Also, there is no total cut-off point. As the score increases, i.e., as it approaches 28 points, it is concluded that the individual is committed to the Mediterranean lifestyle [18]. According to Cemali et al. (2024), the validity and reliability of the MEDLIFE scale in Turkish were assessed, confirming its applicability as a scale [19].

### 2.6. Anthropometric Measurements and Body Composition Analysis

All anthropometric measurements, including body height, body weight, neck, waist, and hip circumferences, as well as body composition analyses and body fat percentage assessments, were conducted by a researcher assigned to this study. Additionally, the waist-to-hip ratio and body mass index (BMI) were calculated. The body weight and body fat composition of the study participants were measured using a Tanita BF-350 BIA device. Measurements were conducted with participants wearing light clothing, free of accessories (e.g., jewelry and watches), and barefoot. Prior to weighing, participants were instructed to schedule the measurement at least five days before or after their menstrual period to reduce the risk of edema-related body composition analysis errors and to use the restroom beforehand. Height was measured with participants standing barefoot, with their feet together, and positioned so that the lower border of the eye socket and the ear canal were aligned on the same horizontal plane, ensuring the head was in the Frankfort plane position. Waist circumference was measured using a non-stretchable tape with participants standing straight, with their arms at their sides and their feet together. The measurement was taken at the midpoint between the lowest rib and the iliac crest, while accounting for breathing status. BMI was calculated using the World Health Organization (WHO) classification (2024), dividing body weight (kg) by the square of height (m). Neck circumference was measured just below the laryngeal prominence (Adam’s apple) using a non-stretchable tape, with measurements greater than 34 cm considered indicative of an increased risk of obesity [20,21].

### 2.7. Analysis and Interpretation of Data Statistical Analysis

The Statistical Package for the Social Sciences (SPSS) version 24.00 was used. While the frequency (n) and percentage (%) distribution of qualitative variables were calculated, arithmetic means ($\bar{X}$), standard deviation (SD), and minimum, and maximum values were determined for quantitative variables. Kolmogorov–Smirnov’s test was used to check normality. As not all of the data were suitable for normality, the Spearman Correlation Test was used to determine correlations between two quantitative variables. *p* < 0.05 was accepted as being statistically significant.

## 3. Results

A total of 120 volunteer women previously diagnosed with HT participated in the study. The mean age of the participants was 37.49 ± 7.47 years; 73.3% of the participants were married; 23.3% were primary or high school graduates; 47.5% had undergraduate degrees; and 29.2% had postgraduate degrees. Additionally, 12.5% of the participants were unemployed, 39.2% were civil servants, and 48.3% were private sector employees. In our study, 65.8% of the individuals did not smoke and 44.2% did not drink alcohol. Additionally, 71.7% of the participants who consumed alcohol drank red wine (Table S1).

Table 1 shows the BMI, waist circumference and waist–hip ratio classification of the participants. Accordingly, it was determined that 27.5% of the participants were obese, 26.7% were pre-obese, and 45.8% were in the normal weight classification. In the waist circumference classification, 31.7% were in the very high health risk group. According to the waist–hip ratio classification of the participants, 80.8% were found to be in the normal range. The average body fat percentage of the participants was measured as 32.51 ± 8.97%. The neck circumference was 33.32 ± 2.50 cm. The waist circumference was 84.75 ± 13.7 cm. The BMI was measured as 26.84 ± 6.10 kg/m^2^ and the hip circumference was measured as 107.47 ± 12.4 cm.

The participants’ compliance with the Mediterranean diet is stated in Table 2. While the average MEDAS score of the participants was observed to be 7.80 ± 1.93 points, it was found that 27.5% had a low, 35% had a moderate and had a 37.45% high level of compliance with the Mediterranean diet. It was calculated that the participants received a score of 8.99 ± 2.65 points for compliance with food consumption, 4.40 ± 1.39 for eating habits, and 3.06 ± 1.20 for activities of daily living, which are the sub-scores of the MEDLIFE score, and it was seen that the total MEDLIFE score was 16.41 ± 3.74.

Prior to the evaluation of the correlation between MEDAS, MEDLIFE scores and anthropometric measurements, an independent sample *t*-test was applied to confirm the effects of medication usage on the MEDAS and MEDLIFE. There was no statistical significance between these variables (Table S2). Table 3 shows the correlations between the participants’ anthropometric measurements and the MEDLIFE total score and subgroup scores and the MEDAS score. Accordingly, it was observed that there were negative correlations between the participants’ body weights and the MEDLIFE total score, Mediterranean dietary habits and physical activity rest, social habits, and conviviality subgroup scores (*p* < 0.05). As the participants’ body weights increased, these scores were negatively affected and decreased. This situation was also valid for BMI, hip circumference, waist circumference, and body fat ratios (*p* < 0.05). On the other hand, an increase in neck circumference caused a decrease in the MEDLIFE scale, Mediterranean dietary habits and physical activity, rest, social habits and conviviality subgroup scores (*p*: 0.009). No significant correlation was observed between the participants’ MEDAS scores and any of the measured anthropometric measurements.

## 4. Discussion

In this study, the relationship between MEDAS and MEDLIFE scores and obesity-related anthropometric measurements in women diagnosed with HT living in the northern part of the Mediterranean island of Cyprus was examined. A strong association was identified between the total and most of the subgroup scores of MEDLIFE and anthropometric measurements. Due to limited data regarding this subject with a similar sample size, which is the strength of our study, we discussed our results with a limited number of studies.

In the presented study (n: 120; 19–54 years old), among the HT patients, 26.7% were classified as pre-obese and 27.5% as obese (Table 1). Similarly, a study (n: 34) by Koszowska et al. (2019) revealed that 35% of women diagnosed with HT were pre-obese [22]. A study conducted by Malczyk et al. (2021) involving 47 HT patients (20–65 years old) found that 83.3% of participants were classified as pre-obese and 16.7% as obese [8]. The average BMI of participants was 26.84 ± 6.10 in this study (Table 1). In a recent study by Klobučar et al. (2024), in which 93.9% of the participants were women (19–72 years old), it was similarly reported that the average BMI of participants diagnosed with HT was 28.3 kg/m^2^ [7]. In our current study, 58.3% of women had a body fat ratio above the ideal range and were therefore determined to be obese. It should be noted that 31.7% of HT women had a waist circumference higher than 88 cm, making them part of the high-risk group, and this range is quite high. Moreover, the average neck circumference of individuals with HT was measured as 33.32 cm. On the other hand, 62.5% of women were in the risk group according to neck circumference (Table 1). A study of women diagnosed with HT (n: 47, 20–65 years old) had similar results and 44.7% of the participants had an excessive body fat ratio [8]. Another study involving 53 HT patients whose mean age was 44.6 years old reported increased waist circumference [23]. Park et al. (2024) conducted a cross-sectional study in Korea involving 8198 patients (40–80 years old). In this study, 5% of the participants had a history of thyroid disease, and an increased neck circumference was found to be particularly common in pre-menopausal women, similarly to the findings of our current study [24].

In this study, the average MEDAS score of the participants was 7.80 ± 1.93 points, and 72.5% of women diagnosed with HT had a moderate or high level of compliance with the Mediterranean diet. No significant correlation was found between the participants’ MEDAS scores and any of the measured anthropometric variables (Table 2 and Table 3). In a recent study conducted by Jureško et al. (2024) on 4620 thyroid gland patients (42–66 years old), it was reported that the rare of compliance with the Mediterranean diet was 19.8% and the total score was determined to be 10 out of 24 on average [25]. A study by Zupo et al. (2020) involving 324 pre-obese and obese individuals (14–72 years old) revealed that BMI and waist circumference values were higher in individuals with Mediterranean diet scores below seven. These findings suggest that increased adherence to the Mediterranean diet may be associated with a lower BMI and better metabolic anthropometric indicators [12]. In a study by Ulker et al. (2024), after a twelve-week Mediterranean diet intervention in HT patients (n: 40, 18–65 years old), significant decreases were observed in body weight, BMI, and body fat percentage in the intervention group compared to the control groups [26]. In another study conducted by Shady et al. (2024), a 6.78% decrease in BMI was observed as a result of the Mediterranean diet applied to 40 HT women for 12 weeks [27].

As shown in Table 3, an increased total MEDLIFE score and increased scores in the subgroups of Mediterranean dietary habits, physical activity, rest, social habits, and conviviality scores were associated with decreased obesity-related anthropometric measurements such as body weight, BMI, and hip and neck circumferences (except for the total MEDLIFE score) (*p* < 0.05, Table 3). In the current literature, no studies have aimed to determine the association between MEDLIFE score and anthropometric measurements in HT patients. That is why this paragraph includes studies conducted in healthy populations. Hershey et al. (2021) conducted a study involving 239 participants (mean age 47 years old). They reported that a decrease in the total MEDLIFE score caused an increase in abdominal obesity risk [28]. Another study in Spain, which involved 11,091 adults, revealed that a lower MEDLIFE score was associated with higher risk of abdominal obesity [29]. Also, in the study by Kamińska et al. (2023), Mediterranean lifestyle changes were applied to HT women for 10 weeks, and significant decreases were recorded in waist circumference and BMI during this period [14].

## 5. Conclusions

In conclusion, beyond the Mediterranean diet, adherence to the Mediterranean lifestyle may effectively reduce the risk of pre-obesity and obesity in patients with HT. The Mediterranean lifestyle may have beneficial effects on the quality of life of patients with HT. Our study highlights the importance of conducting anthropometric assessments and, when necessary, implementing lifestyle interventions, as individuals with HT often have a high prevalence of pre-obesity and obesity. However, there were some limitations. The sample size was calculated as 138 for this study. Participation was voluntary and only 120 patients participated. Moreover, as a cross-sectional study, the presented study utilized self-reported data, which are subject to recall bias. Also, the determination of nutritional habits, patterns and physical activity behaviors was limited to the MEDLIFE scale. Moreover, these results are limited to the Northern part of Cyprus; thus, generalizing the results may not be possible.

## Figures and Tables

**Table 1:** Classification and distribution of the participants’ BMI, waist circumference, waist–hip ratio, and neck circumference (*n*: 120).

	n (%)		
BMI (kg/m^2^)	Normal (18.5–24.9)		55 (45.8)
	Pre-obesity (25.0–29.9)		32 (26.7)
	Obesity (>30.0)		33 (27.5)
	Total		120 (100)
Waist circumference (cm)	Low Health Risk (<80)		55 (45.8)
	Moderate Health Risk (80–88)		27 (22.5)
	High Health Risk (>88)		38 (31.7)
	Total		120 (100)
Waist–Hip Circumference	Low Health Risk (<85)		97 (80.8)
	High Health Risk (>85)		23 (19.2)
	Total		120 (100)
Neck Circumference (cm)	Low Health Risk (<34)		45 (37.5)
	High Health Risk (>34)		75 (62.5)
	Total		120 (100)
Body Fat (%)	<25		25 (20.8)
	25–30		25 (20.8)
	>30		70 (58.3)
	Total		120 (100)
	Minimum	Maximum	$\bar{X}$ ± SD
Height (cm)	142.0	182.0	163.06 ± 6.68
Body Weight (kg)	45.2	124.3	71.52 ± 16.90
Body Mass Index (kg/m^2^)	18.70	45.10	26.84 ± 6.10
Hip Circumference (cm)	82.0	149.0	107.47 ± 12.4
Waist Circumference (cm)	63.00	130.00	84.75 ± 13.70
Neck Circumference (cm)	28.00	41.80	33.32 ± 2.50
Body Fat Percentage (%)	14.10	50.50	32.51 ± 8.97

**Table 2:** Classification and distribution of participants’ average MEDAS scores and levels of adherence to the Mediterranean diet and quality of life (*n* = 120).

			$\bar{X}$ ± SD (min–max)
MEDAS Score			7.80 ± 1.93 (0.0–14.0)
			n (%)
Mediterranean food consumption	Low		33 (27.5)
	Moderate		42 (35.0)
	High		45 (37.5)
	Total		120 (100)
MEDLIFE scores	Minimum	Maximum	$\bar{X}$ ± SD
Mediterranean food consumption	2	14	8.99 ± 2.65
Mediterranean dietary habits	0	6	4.40 ± 1.39
Physical activity, rest, social habits and conviviality	0	5	3.06 ± 1.20
Total MEDLIFE score	8	26	16.41 ± 3.74

**Table 3:** Correlation distributions of participants’ anthropometric measurements with MEDLIFE, its subgroups, and MEDAS scores (*n*: 120).

		MEDLIFE				MEDAS
		Mediterranean Food Consumption	Mediterranean Dietary Habits	Physical Activity, Rest, Social Habits and Conviviality	Total Score	Total Score
Body weight (kg)	r	−0.146	−0.238	−0.289	−0.301	−0.073
	p_1_	0.112	0.009 **	0.001 **	0.001 **	0.429
BMI (kg/m^2^)	r	−0.083	−0.217	−0.236	−0.231	−0.051
	p_1_	0.356	0.017 *	0.010 *	0.011 *	0.582
Hip Circum. (cm)	r	−0.141	−0.233	−0.276	−0.299	−0.123
	p_1_	0.124	0.011 *	0.002 **	0.001 **	0.182
Waist Circum. (cm)	r	−0.127	−0.238	−0.299	−0.302	−0.122
	p_1_	0.168	0.009 *	0.001 **	0.001 **	0.186
Neck Circum. (cm)	r	−0.021	−0.181	−0.242	−0.169	−0.007
	p_1_	0.816	0.047 *	0.008 **	0.066	0.942
Body Fat (%)	r	−0.097	−0.282	−0.357	−0.317	−0.070
	p_1_	0.291	0.002 **	<0.001 **	<0.001	0.445

p_1_: Spearman’s correlation test. * Correlation is significant at the 0.05 level (2-tailed). ** Correlation is significant at the 0.01 level (2-tailed).

## Data Availability

The dataset used in this study is available on request.

## Supplementary Materials

The following supporting information can be downloaded at: https://www.mdpi.com/article/10.3390/nu17050892/s1; Table S1: Distribution of Participants by Sociodemographic and General Health Characteristics; Table S2: Impact of Thyroid Medication Usage on the MEDAS and MEDLIFE.

## Abbreviations

The following abbreviations are used in this manuscript:

HT	Hashimoto Thyroiditis.
Anti-TPO	Thyroid Peroxidase.
Anti-TG	Thyroglobulin
TSH	Thyroid-stimulating hormone.
T_4_	Thyroxine.
MEDLIFE	Mediterranean Lifestyle Index.
MEDAS	Mediterranean Diet Adherence Screener.
WHO	World Health Organization.
SPSS	Statistical Package for the Social Sciences.
SD	Standard Deviation.
