# Supplementary material for: Association Between Anthropometric Measurements and Mediterranean Lifestyle in Women Diagnosed with Hashimoto’s Thyroiditis: Data from the Mediterranean Region
Source: Nutrients. 2025 Mar 3;17(5):892. doi: 10.3390/nu17050892 (PMC11901783; doi:10.3390/nu17050892)
Supplement: Supplementary file 1 [file nutrients-17-00892-s001.zip › nutrients-3504435-supplementary.pdf]

**Table S1.** Distribution of Participants by Sociodemographic and General Health Characteristics (n: 120).

| | | X±SD (min-max) |
|---|---|---|
| Age (year) | | 37.49±7.47 (19-54) |
| | | **n (%)** |
| Marital Status | Married | 88 (73.3) |
| | Single | 32 (26.7) |
| | **Total** | **120 (100)** |
| Educational Status | Primary School-High School | 28 (23.3) |
| | Undergraduate/College | 57 (47.5) |
| | Postgraduate | 35 (29.2) |
| | **Total** | **120 (100)** |
| Profession | Student/ Housewife | 15 (12.5) |
| | Civil servant | 47 (39.2) |
| | Private sector employee | 58 (48.3) |
| | **Total** | **120 (100)** |
| Smoking | Yes | 41 (34.2) |
| | No | 79 (65.8) |
| | **Total** | **120 (100)** |
| Alcohol Consumption | Yes | 67 (55.8) |
| | No | 53 (44.2) |
| | **Total** | **120 (100)** |
| Alcohol Type (n: 53) | Red Wine | 38 (71.7) |
| | Others | 15 (28.3) |
| | **Total** | **53 (100)** |
| **General Health Condition** | | **n (%)** |
| Obesity Comorbidity | Yes | 39 (32.5) |
| | No | 81 (83.3) |
| | **Total** | **120 (100)** |
| Diagnosis of Hashimoto Thyroiditis in the Family | Yes | 43 (35.8) |
| | No | 77 (64.2) |
| | **Total** | **120 (100)** |
| | | **X±SD (min-max)** |
| What age were you diagnosed with Hashimoto's thyroiditis? (year) | | 29.9±8.49 (8-53) |
| | | **n (%)** |
| Year that you were diagnosed with Hashimoto's thyroiditis? | <5 year | 50 (41.7) |
| | 5-10 year | 26 (21.7) |
| | >10 year | 44 (36.7) |
| | **Total** | **120 (100)** |
| Daily Thyroid Hormone Intake | Yes | 58 (48.3) |
| | No | 62 (51.7) |
| | **Total** | **120 (100)** |

**Table S2.** Impact of Thyroid Medication Usage on the MEDAS and MEDLIFE (n: 120).

| | **Thyroid Medication Usage Status** | | |
|---|---|---|---|
| | **User (n: 75)** | **Non-User (n: 45)** | |
| | **X̄±SD** | **X̄±SD** | **p** |
| **MEDAS** | 7.74±1.88 | 7.88±2.03 | 0.698 |
| **MEDLIFE** | 16.28±3.80 | 16.64±3.68 | 0.608 |
| ***MEDLIFE Subgroups*** | | | |
| Mediterranean Food Consumption | 9.13±2.65 | 8.75±2.65 | 0.452 |
| Mediterranean Dietary Habits | 4.25±1.50 | 4.64±1.17 | 0.139 |
| Physical Activity, Rest, Social Habits and Conviviality | 3.01±1.23 | 3.15±1.16 | 0.534 |

p: Independent sample t-test
